# C-Reactive Protein at Hospital Discharge and 1-Year Mortality in Acute Infective Endocarditis: A Prospective Observational Study

**DOI:** 10.3389/fcvm.2021.706684

**Published:** 2021-08-09

**Authors:** Yaowang Lin, Jie Chen, Bihong Liao, Weijie Bei, Yongshun Wang, Xin Sun, Jie Yuan, Shaohong Dong

**Affiliations:** ^1^Department of Cardiology, Shenzhen Cardiovascular Minimally Invasive Medical Engineering Technology Research and Development Center, Shenzhen People's Hospital Second Clinical Medical College, Jinan University, Shenzhen, China; ^2^First Affiliated Hospital, Southern University of Science and Technology, Shenzhen, China

**Keywords:** C-reactive protein, acute infective endocarditis, all-cause death, paravalvular abscess, rehospitalization

## Abstract

**Background:** An accurate biomarker at hospital discharge is needed to identify patients with acute infective endocarditis (IE) who are at high risk of mortality. This prospective observational study evaluated the prognostic value of C-reactive protein (CRP).

**Methods:** Patients with acute IE (*n* = 343) and hospitalized at 2 university-affiliated medical centers from January 2014 to December 2019 were enrolled. Patients were categorized as having low or high CRP (*n* = 217 and 126, respectively) at hospital discharge according to the optimal cutoff (CRP = 6.5 mg/L) determined via receiver-operating characteristic curve analysis. The primary endpoint was all-cause death, from hospital discharge to 1 year. The secondary endpoint was the cumulative rate of rehospitalization or paravalvular abscess at 1 year.

**Results:** At the 12-month follow-up, the mortality rate of the high-CRP group (21.43%) was significantly higher than that of the low-CRP group (2.76%, log-rank *P* < 0.0001). The multivariate regression analysis indicated that the high-CRP group had a higher excess mortality hazard risk (*HR* = 4.182; 95% CI: 2.120, 5.211; *P* < 0.001). The cumulative 1-year incidence of paravalvular abscess of the high-CRP group (11.90%) was significantly higher than that of the low-CRP (5.07%; *P* = 0.022). The cumulative rate of heart rehospitalizations of the 2 groups were similar (18.25% cf. 14.29%, *P* = 0.273).

**Conclusion:** For hospitalized patients with acute IE, a high CRP at discharge suggests a poor prognosis for 1-year mortality and paravalvular abscess.

## Introduction

IE is an important source of mortality and morbidity, entailing expensive treatments, long-term hospitalization and challenging interventions. Despite standardized medical therapy or surgical treatments, its poor prognosis is reflected by 8–24% in-hospital mortality that rises up to 18–37% during the first year after the IE episode (1). Moreover, temporal trend analyses describe an overall increasing incidence of IE over the past 20 years (2). The lack of prognosis biomarkers represents a challenge for both short and long-term risk stratification in IE. Accordingly, there should be a means to identify those patients at discharge who have an elevated risk of mortality.

Despite being a non-specific biomarker, CRP has proven to improve risk stratification in different forms of cardiovascular disease. Importantly, CRP at discharge was associated with an excess of 1-year mortality after acute decompensated heart failure hospitalization (3) and might be a long-term prognosis marker in IE. At hospital admission, C-reactive protein (CRP) is a putative predictor of adverse prognosis in patients with IE (4, 5), but data on its prognostic value after at discharge post-treatment is limited. Heiro et al. (5) reported that a rise in CRP of 50 mg/L was associated with a higher risk of death, both at 2 weeks (*OR* = 1.5, 95% CI, 0.97–2.3; *P* = 0.068) and 4 weeks after admission (*OR* = 2.3, 95% CI, 1.2–4.4; *P* = 0.009). As a response to chronic inflammation, the CRP level thus may be a useful indicator for patients with IE leaving the hospital.

To assist in determining a prognosis for patients with IE leaving the hospital, the present observational study is the first to assess an association between CRP level at discharge and clinical outcomes at 1 year, for patients with acute IE.

## Materials and Methods

### Patient Enrollment

The study was designed as a prospective observational trial and conducted at 2 medical centers (Shenzhen People's Hospital and Guangdong General Hospital) in China. The minimum sample size of each group (*n* = 69, alpha = 0.05, beta = 0.20, based on a frequency of 1-year mortality of 15% in the high-CRP group and 0.2% in the low-CRP group, with 1:1 sampling), was calculated using the chi-squared test in PASS 15 software (6). The Research Ethics Committee of Shenzhen People's Hospital approved the protocol (LL-KY-2014009). All patients provided informed consent before their enrollment.

**Figure 1:**
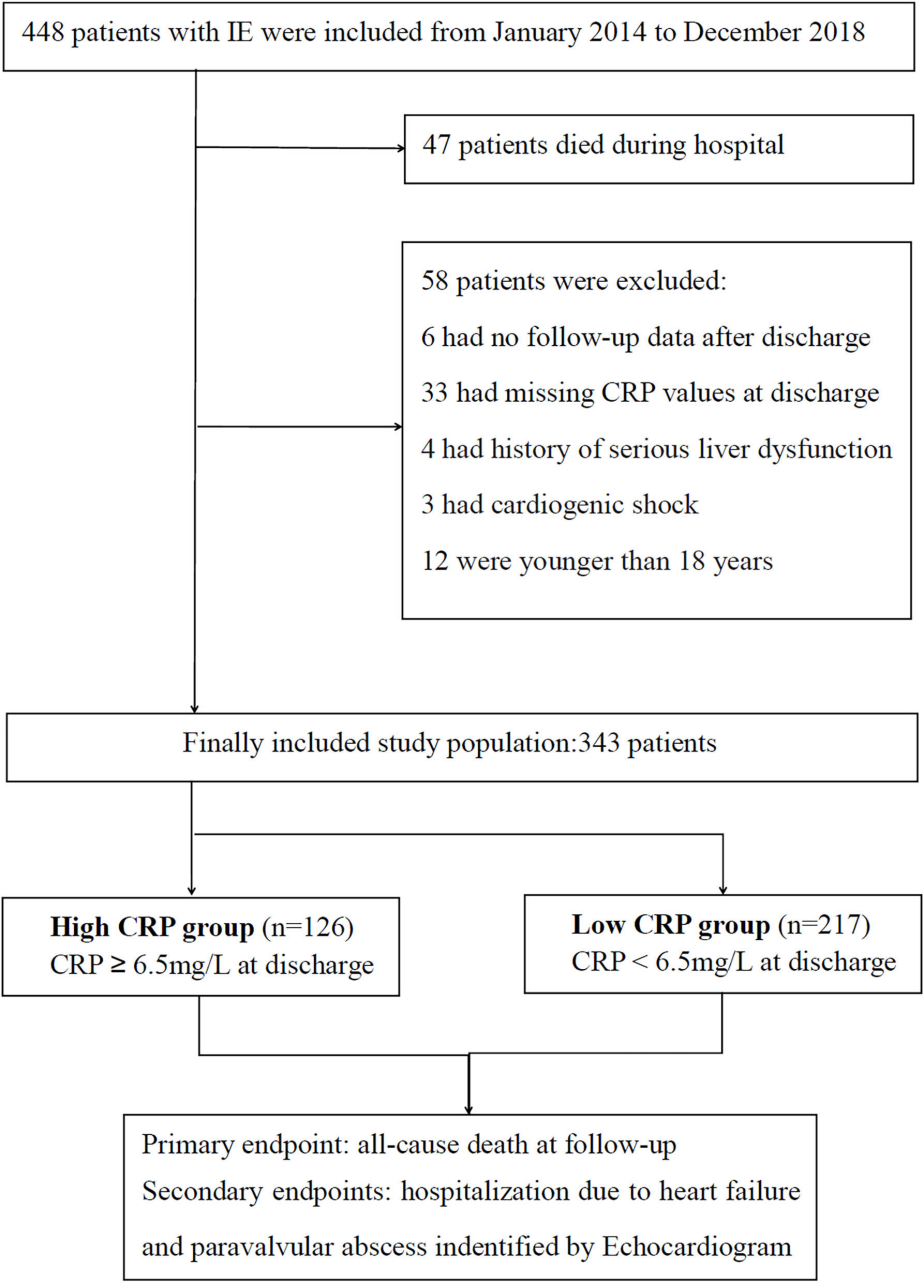
Schematic illustration of patient inclusion and data analysis.

Based on the modified Duke criteria (7), 448 patients with acute IE were screened from January 2014 to December 2019 (Figure 1). Patients were excluded from this analysis for the following reasons: death during hospital stay (*n* = 47); missing CRP values at discharge (*n* = 33); younger than 18 years (*n* = 12); without follow-up data (*n* = 6); serious liver dysfunction, active neoplasm and other inflammatory diseases (*n* = 4); or cardiogenic shock (*n* = 3). Finally, 343 patients with acute IE were included in this study. Surgery was performed when the surgical criteria were met. Conservative drug therapy was initiated in circumstances of extremely poor health, or the patient could not afford the surgical fee.

### Data Collection

The CRP value (normal reference value: 0–5 mg/L) was analyzed by immunoturbidimetry assay, on admission and at discharge. Transthoracic echocardiography was assessed within 24 h of admission. All-cause death was defined as subsequent readmission or mortality during the 1-year follow-up after discharge. Rehospitalization due to heart failure or relapse was defined as worsening heart failure or a repeat episode of IE caused by the same microorganism as the previous episode (within 6 months) (8), if either required intravenous drug therapy. Paravalvular abscess meant the presence of necrotic tissue in the valve annulus or remaining cavity after septic destruction of the valve annulus, as revealed by echocardiogram, transesophageal echocardiography, cardiac computed tomography, or surgery if needed during follow-up.

### Study Endpoints

The primary study endpoint was the incidence of all-cause death at 1-year. The secondary endpoints included the cumulative rate of rehospitalization and paravalvular abscess at 1-year.

### Statistical Analysis

Statistical analyses were performed using SPSS 22.0 software. Data was reported as mean ± standard deviation when normally distributed, quartile range when not normally distributed or count (percentage). Group comparisons of continuous data were conducted using Student's test, analysis of variance (ANOVA), or Kruskal-Wallis test; group comparisons of categorical data were calculated by chi-squared or Fisher's exact test. The optimal cutoff was determined via receiver operator characteristic curve (ROC) analysis. Univariate (crosstabs-relative risk) and multivariate (binary logistic regression) analyses were applied to evaluate the adjusted hazard ratio (HR) of mortality with a 1-year follow-up. The Kaplan-Meier method was applied to evaluate long-term survival. Enhanced predictive accuracy of the CRP-based model and the clinical model was evaluated by net reclassification improvement (NRI) and integrated discrimination improvement (IDI). *P* < 0.05 was considered statistically significant.

## Results

### Characteristics of Patients in the High- and Low-CRP Groups

**Figure 2:**
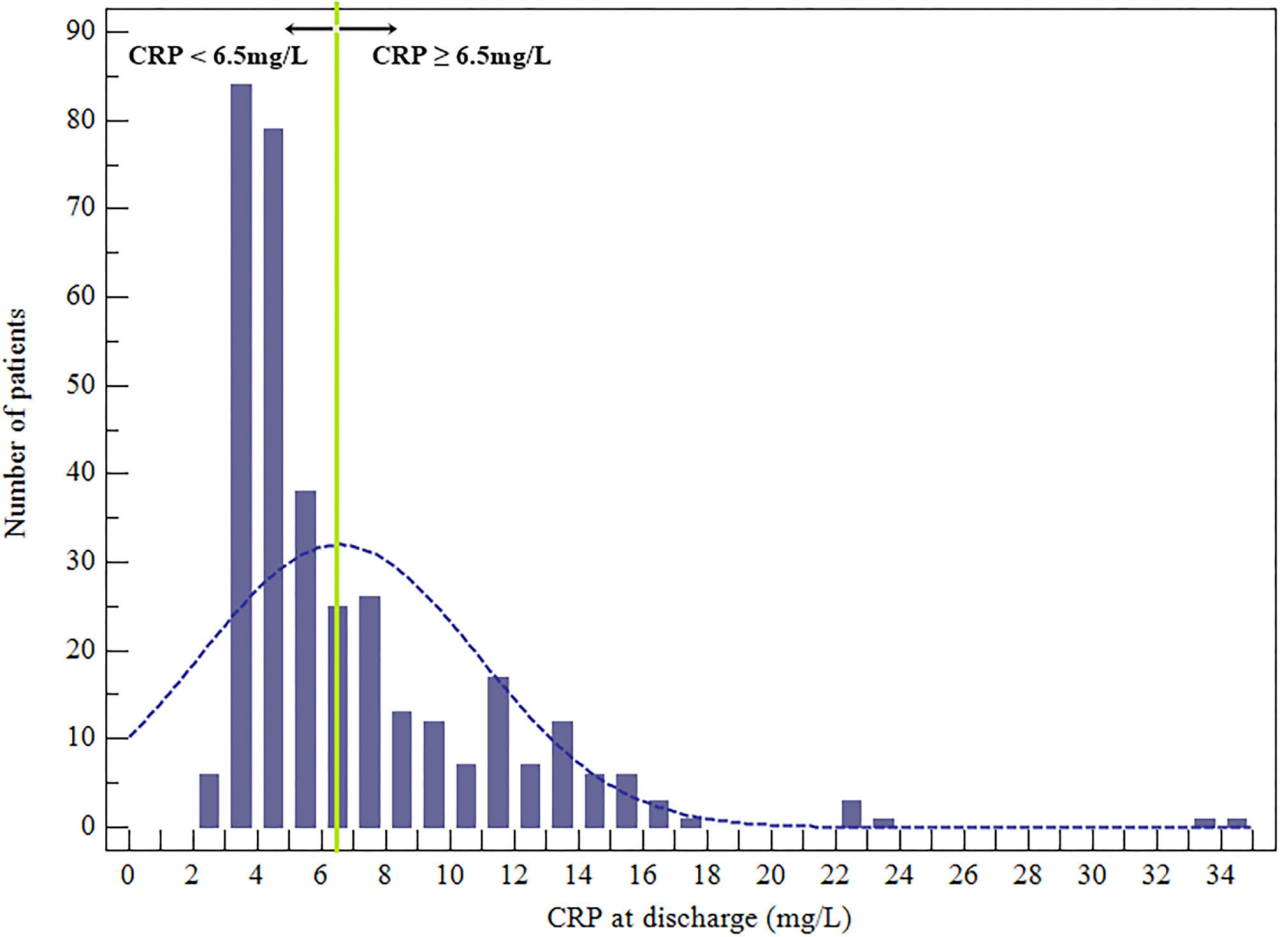
Distribution of the CRP values at hospital discharge.

Overall, there were 343 patient participants (115 women), among whom 33 (9.62%) died during the 1-year follow-up. The CRP levels at discharge were abnormally distributed (Figure 2). According to the CRP value at the time of hospital discharge, according to the optimal cutoff patients were classified as low-CRP (<6.5 mg/L, *n* = 217) or high-CRP (≥6.5 mg/L, *n* = 126). The ages of the low- and high-CRP groups were comparable (45.99 ± 15.83 and 47.76 ± 15.56 y, respectively; *P* = 0.315). Upon admission, troponin I levels were measured in 212 patients (61.81%) of which 81 (64.29%) and 131 (60.37%) were apportioned to high-CRP or low-CRP groups, respectively. Importantly, 44 (54.32%) patients in the high-CRP group and 30 (22.90%) patients in the low-CRP group had elevated troponin I levels (*P* < 0.001; Table 1). Other features that were similar between the groups were the baseline demographics, echocardiography, causative pathogens, laboratory indexes, duration of antibiotic therapy, and hospital stay.

**Table 1:** Baseline clinical characteristics of patients according to CRP group.

	**Low-CRP**	**High-CRP**	***P***
Subjects, *n* (%)	217 (63.26%)	126 (36.73%)	—
Age, years (mean ± SD)	45.99 ± 15.83	47.76 ± 15.56	0.315
Females, *n* (%)	79 (36.41)	36 (28.57)	0.193
AIE, *n* (%)	42 (19.35)	22 (17.46)	—
SIE, *n* (%)	175 (80.65)	104 (82.60)	0.774
Hypertension, *n* (%)	20 (9.22)	13 (10.32)	0.708
Diabetes mellitus, *n* (%)	5 (2.30)	5 (3.97)	0.507
**Affected valve**
Aortic valve, *n* (%)	104 (47.93)	56 (44.44)	0.822
Mitral valve, *n* (%)	105 (48.39)	70 (55.00)	0.090
Triple vale, *n* (%)	25 (11.52)	12 (10.00)	0.719
Multiple-valves, *n* (%)	28 (12.90)	18 (14.286)	0.945
Congenital heart disease, *n* (%)	5 (2.30)	5 (3.97)	0.492
Prosthetic valve, *n* (%)	7 (3.23)	20 (15.87)	*0.001*
Neurological failure (GCS ≤ 12), *n* (%)	12 (5.53)	10 (7.94)	0.426
Stroke, *n* (%)	17 (7.83)	12 (9.52)	0.551
Heart failure, *n* (%)	120 (55.30)	67 (60.87)	0.824
NYHA III–IV, *n* (%)	107 (49.31)	62 (60.48)	0.082
^a^LVEF (%), mean ± SD	62.90 ± 7.75	62.29 ± 9.24	0.717
**Pathogen**
*Staphylococcus aureus, n* (%)	6 (2.76)	5 (3.97)	0.623
*Streptococcus spp, n* (%)	30 (13.82)	21 (16.67)	0.430
Iatrogenic infection, *n* (%)	6 (2.76)	4 (3.17)	0.521
Vegetation size ≥10 mm, *n* (%)	101 (33.80)	56 (55.00)	0.822
Temperature, °C	38.16 ± 1.27	38.85 ± 0.57	0.714
Troponin obtained on admission, *n* (%)	131 (60.37)	81 (64.29)	—
^b^Elevated Troponin I level, *n* (%)	30 (22.90)	44 (54.32)	*0.001*
ESR mm/h on admission, mean ± SD	36.25 ± 33.73	36.95 ± 35.41	0.856
CRP on admission, mean ± SD	23.81 ± 23.41	26.20 ± 23.77	0.364
CRP at discharge, median (IQR)	4 (3.3, 4.9)	10.2 (7.3, 13.0)	*0.000*
Duration of hospital stay, days (mean ± SD)	48.33 ± 12.29	46.52 ± 11.40	0.177
Duration of antibiotic therapy, days (mean ± SD)	45.12 ± 11.29	45.72 ± 12.02	0.844
Surgery treatment, *n* (%)	193 (88.94)	104 (82.54)	0.102
1-year mortality, *n* (%)	6 (2.76)	27 (21.43)	*0.000*
Rehospitalization, *n* (%)	31 (14.29)	23 (18.25)	0.358
Paravalvular abscess, *n* (%)	11 (5.07)	15 (11.90)	*0.032*

a *LVEF cutoff was 54%*.

b *Elevated Troponin I was ≥ 0.01ng/ml. AIE, acute infective endocarditis; ESR, erythrocyte sedimentation rate; GCS, Glasgow Coma Score; LVEF, left ventricular ejection fraction; SIE, subacute infective endocarditis. The meaning of italic value p = 1.0*.

### CRP at Discharge Predicting Mortality at 1-Year and Clinical Outcomes

**Figure 3:**
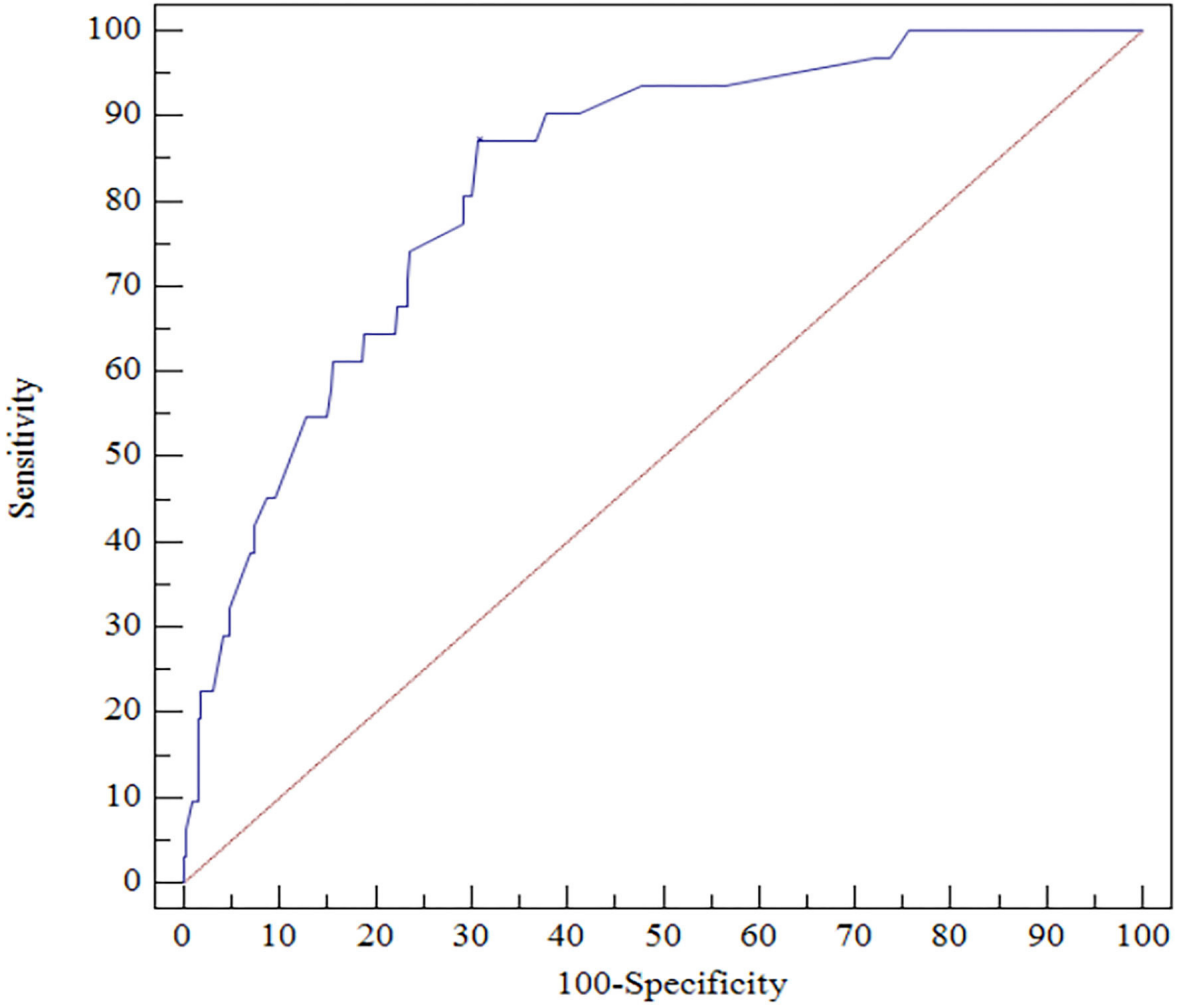
ROC curves for CRP predicting 1-year all-cause death.

The ROC analysis indicated that CRP ≥ 6.5 mg/L at discharge could accurately predict 1-year mortality with a sensitivity of 87.10% and a specificity of 69.23% (area under the ROC curve = 0.827, 95% CI, 0.783–0.865, *P* < 0.0001; Figure 3).

**Figure 4:**
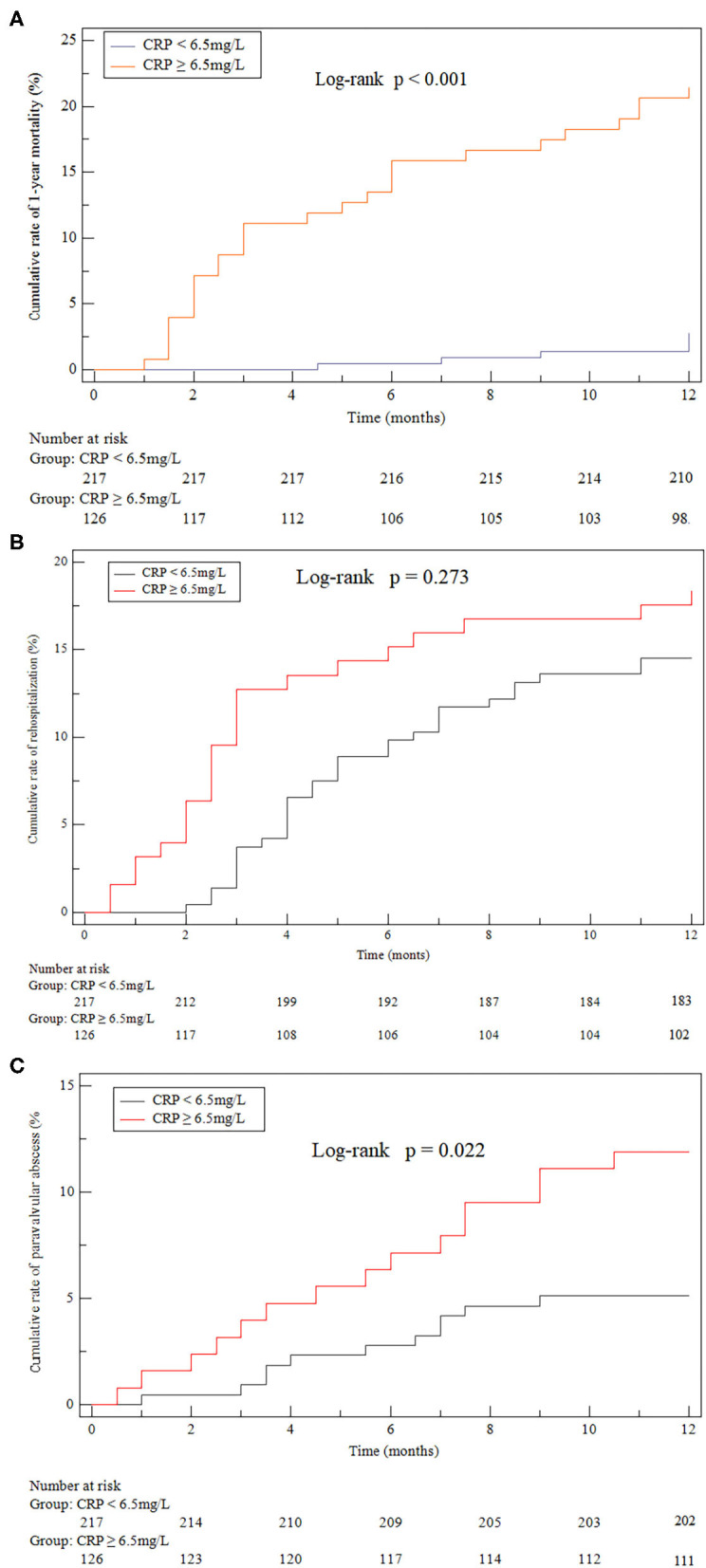
Kaplan-Meier curves. **(A)** all-cause death after discharge (21.43% cf. 2.76%, *P* < 0.001). **(B)** Heart failure hospitalizations after discharge (18.25% cf. 14.29%, *P* = 0.273). **(C)** Paravalvular (11.90% cf. 5.07%, *P* = 0.022) abscess identified by echocardiogram after discharge compared between the high-CRP and low-CRP groups.

Among the 343 patients, 33 (9.62%) died within the follow-up of 12 months, including 27 (27/126, 21.43%) in the high-CRP group and 6 (6/217, 2.76%) in the low-CRP group (*P* < 0.0001, chi-squared test). The Kaplan-Meier analysis showed a lower cumulative survival ratio in patients with high CRP ≥ 6.5 mg/L (*P* < 0.0001; Figure 4A).

According to the univariate logistic regression analysis (crosstabs-relative risk), additional significant indicators included age, diabetes mellitus, heart failure, New York Heart Association (NYHA) III–IV, endocarditis vegetation size ≥10 mm, *Staphylococcus aureus*, troponin, CRP, and surgery. After adjusting for the variables of significance shown by the univariate analysis, the multivariate regression analysis confirmed that CRP at discharge was an independent predictor of 1-year mortality (*HR* = 4.182; 95% CI: 2.120, 5.211, *P* < 0.000; concordance index = 0.81, 95% CI: 0.65–0.86; Table 2). Compared to CRP-based model, the clinical model did not showed the enhanced predictive accuracy with regard to NRI (0.002, *p* = 0.532) and IDI (0.047, *p* = 0.470).

**Table 2:** Univariate analyses and multivariate Cox proportional hazard of factors associated with 1-year mortality.

			**Univariate**		**Multivariate**	
	**Survivors**	**All-cause death**	**HR (95% CI)**	***P***	**HR (95% CI)**	***P***
Subjects, *n* (%)	310 (90.37%)	33 (9.62%)	—	—	—	—
Age, years (mean ± SD)^a^	46.01 ± 15.54	52.54 ± 16.49	1.280 (0.66–4.52)	*0.023*	1.112 (1.152–4.421)	*0.020*
Males, *n* (%)	209 (72.89)	22 (66.67)	0.953 (0.425–2.040)	0.543		
AIE, *n* (%)	52 (17.78)	10 (19.05)	1.452 (0.025–2.140)	0.471		
Hypertension, *n* (%)	28 (9.33)	5 (15.15)	1.799 (0.644–5.027)	0.263		
Diabetes mellitus, *n* (%)	7 (3.23)	3 (9.091)	2.324 (1.063–6.36)	*0.034*	2.122 (1.022, 5.545)	*0.066*
**Affected valve**						
Aortic valve, *n* (%)	143 (43.11)	15 (61.90)	1.081 (0.491–2.112)	0.962		
Mitral valve, *n* (%)	153 (52.0)	17 (42.86)	1.156 (0.557–2.396)	0.698		
Triple vale, *n* (%)	32 (12.44)	5 (4.76)	1.568 (0.564–4.359)	0.388		
Multiple-valves, *n* (%)	20 (8.89)	2 (9.52)	1.01 (0.4–2.56)	0.761		
Congenital heart disease, *n* (%)	37 (12.44)	7 (14.29)	1.964 (0.797–4.845)	0.143		
Neurological failure^b^, *n* (%)	13 (6.22)	3 (19.05)	2.285 (0.616–5.97)	0.217		
Stroke, *n* (%)	26 (8.89)	3 (28.57)	1.089 (0.311–3.081)	0.89*5*		
Heart failure, *n* (%)	158 (46.67)	26 (76.19)	3.293 (1.506–8.476)	*0.028*	2.0.967 (1.355, 7.220)	*0.046*
NYHA III–IV, *n* (%)	134 (42.)	21 (61.90)	2.299 (1.092–4.837)	*0.003*	2.014 (1.243, 3.610)	*0.001*
LVEF^c^, *n* (%)	62.72 ± 9.39	61.90 ± 8.56	0.39 (0.14–1.11)	0.78		
Vegetation size ≧10 mm, *n* (%)	131 (40.89)	23 (61.90)	3.090 (1.422–6.715)	*0.004*	2.224 (1.055, 4.713)	*0.033*
*Staphylococcus aureus, n* (%)	8 (2.58)	3 (9.09)	1.870 (1.212–3.621)	*0.001*	1.543 (1.052, 2.710)	*0.001*
*Streptococcus spp, n* (%)	30 (14.84)	5 (15.15)	1.011 (0.810–2.311)	0.812		
Elevated Troponin I level, *n* (%)	186 (60.00)	26 (78.79)	*3.982 (1.850–8.510)*	*0.001*	*3.110 (1.660–8.322)*	*0.001*
ESR mm/h^d^, mean ± SD	34.15 ± 34.80	36.76 ± 34.30	1.032 (0.836–2.110)	0.835		
CRP on admission, mean ± SD	24.50 ± 23.98	26.88 ± 19.97	1.122 (1.006–3.031)	0.754		
CRP at discharge, median (IQR)	4.5 (3.6, 7.0)	11 (7.1, 14)	4.730 (2.236–5.870)	*0.000*	4.182 (2.120, 5.211)	*0.000*
Duration of hospital stay, days (mean ± SD)	46.64 ± 12.25	47.99 ± 9.34	1.001 (0.523–2.000)	0.925		
Surgery treatment, *n* (%)	279 (89.29)	18 (47.62)	0.133 (0.061–0.291)	*0.000*	0.174 (0.086, 0.341)	*0.000*

a *Age cutoff was 43 years old.*;

b *GCS ≤ 12*;

c *LVEF cutoff was 54%*;

d *ESR cutoff was 44 mm*.

*AIE, acute infective endocarditis; ESR, erythrocyte sedimentation rate; GCS, Glasgow coma Score; SIE, subacute infective endocarditis; LVEF, left ventricular ejection fraction*.

*The meaning of italic values are “reference”*.

Twenty-seven patient participants suffered from paravalvular abscess during the 1-year follow-up. Overall, paravalvular abscess occurred in 15 (55.56%) patients due to a relapse and in 9 (33.33%) patients due to a new IE. Three (11.11%) patients suffered from paravalvular abscess without a confirmed reason. The diagnosis of paravalvular abscess was made by transesophageal echocardiography in 17 (62.96%) patients and by cardiac computed tomography in 4 (14.81%) patients. In the remaining 6 (22.22%) patients the diagnosis was made at surgery. The cumulative 1-year incidence of paravalvular abscess of the high-CRP group (11.90%) was significantly higher than that of the low-CRP group (5.07%; *P* = 0.022, Figure 4C). The groups were statistically similar with regard to the cumulative rate of rehospitalizations due to heart failure and relapse (18.25% cf. 14.29%, *P* = 0.273, Figure 4B).

## Discussion

This study is the first clinical trial to evaluate the CRP level at hospital discharge for predicting 1-year mortality in patients with acute IE. It was found that CRP ≥ 6.5 mg/L was strongly predictive of 1-year mortality at discharge from the index hospitalization, and in addition was associated with a high risk for paravalvular abscess by the 1-year follow-up. However, the groups above and below this CRP cutoff showed no significant difference in risk of hospitalization due to heart failure.

Despite the development of new antibiotics and modern surgical techniques in the past few decades, the mortality of patients with acute IE after discharge is still very high (9, 10). Previous studies have described elevated troponin as a surrogate for increased likelihood of mortality in patients with IE (11–13). A recent meta-analysis reported that patients with elevated troponin suffered a higher rate of in-hospital mortality (*OR* = 5.96, *P* < 0.0001), 1-year mortality (*OR* = 2.67, *P* = 0.002), surgery rates (*OR* = 2.34, *P* = 0.0008), and more frequent complications including central nervous system events (*OR* = 8.85, *P* < 0.0001) and cardiac abscesses (*OR* = 4.96, *P* = 0.0008) (14). Elevated troponin on admission was associated with 1-year mortality risk in this study. Other biochemical markers such as B-Type Natriuretic Peptide, D-dimer, and procalcitonin are reliable prognostic biomarkers associated with adverse outcomes (15–17). Recently, IL-5 and CCL4 were found to add prognostic value beyond CRP levels for in-hospital mortality in IE (18). Multimarker panels might be a useful tool to assess short and long-term prognosis in IE.

CRP is an acute-phase inflammatory serum protein that rapidly responds to infection, and has proved valuable for early risk stratification of sepsis-suspected mortality in patients (19–22). Mohanan et al. (4) found that, in patients with IE, CRP >40 mg/L in the first 3 days of admission was a strong predictor of in-hospital death (*OR* = 8.29, 95% CI: 1.39–49.41, *P* = 0.021); and 6-month mortality (*OR* = 5.55; 95% CI 1.65–18.72, *P* = 0.006). In an evaluation of left-sided native valve endocarditis, Verhagen et al. (23) reported that after 1 week of treatment, CRP >122 mg/L, or a slow percentage decline in CRP level, were indicators of serious infectious complications or death within 3 months (*OR* = 10.5, = 1.1, respectively). Wei et al. (24) showed that elevated CRP (>17.8 mg/L) was associated with in-hospital death in patients with blood culture-negative IE (*OR* = 2.41, 95% CI: 1.06–5.51, *P* = 0.037).

Unfortunately, these previous studies focused only on the efficacy of biochemical markers at admission or early hospitalization. However, a recent prospective cohort study suggested that serum CRP concentration fell significantly faster in patients with an uncomplicated recovery from IE compared with those with complications needing cardiac surgery (2.5% cf. 17.24%) or experiencing death (nil cf. 6.0%) within 3 months (5). Similarly, the present study showed that, for patients with IE, CRP ≥ 6.5 mg/L at discharge was a simple predictor of 1-year mortality caused by underlying inflammation that could not be controlled by antibiotic therapies. For most patients, escalating inflammation or uncontrolled infection caused hemodynamic collapse by increasing oxygen demand and surge (3, 25). Therefore, elevated CRP at discharge may reflect a persistent and chronic inflammatory response, even for patients who received optimal treatment or adequate antibiotic treatment.

The present study determined that CRP ≥ 6.5 mg/L at discharge was associated with an excess risk for paravalvular abscess within 1 year, but not with risk of heart failure hospitalization. The mortality and morbidity rates of patients with active IE complicated with paravalvular abscess remain high after surgery, with 1-year mortality rates of 25 to 50% in patients with aortic periannular abscess (26, 27). Infection often occurs in the annulus or tissue of the prosthetic valve, and extends to the myocardium, eventually leading to valve rupture and paravalvular abscess (28). Accordingly, CRP, a reflection of systemic inflammatory response, is a significant predictor of infective complications and paravalvular abscess (29). However, in the present study the risk of rehospitalization for heart failure at discharge was not significantly higher in the high-CRP (≥6.5 mg/L) relative to the low-CRP group. Additionally, the baseline left ventricular ejection fraction, or NYHA class ≥ 2, did not differ significantly between these groups (Table 1). These findings were similar to that of previous studies, and suggests that elevated CRP levels at discharge are not associated with the severity of cardiac dysfunction or symptoms in chronic heart failure (25, 30).

It should be noted that in the present study, no comparison was conducted between different treatments (surgery or not) and clinical outcomes. In addition, while we considered the CRP level only on the date of discharge, in individuals CRP varies over time. Our next study will monitor CRP concentrations at various timepoints during hospitalization. The incidence of pathogens reported in this study was low and additional information on the epidemiology was lacking. Finally, in the present study only a single CRP cutoff was considered, although a scoring system and the evaluation of additional predictive factors may be more informative.

## Conclusion

CRP at discharge is a convenient prognostic tool for patients with acute IE. A CRP ≥ 6.5 mg/L at hospital discharge was significantly associated with mortality during the first year.

## Data Availability Statement

The raw data supporting the conclusions of this article will be made available by the authors, without undue reservation.

## Ethics Statement

The studies involving human participants were reviewed and approved by The Research Ethics Committee of Shenzhen People's Hospital approved the protocol. The patients/participants provided their written informed consent to participate in this study.

## Author Contributions

YL, YW, and XS collected, analyzed, and wrote this manuscript. BL, WB, and JC assisted in conducting the study. JY and SD were the principal investigators. All authors contributed to the article and approved the submitted version.

## Conflict of Interest

The authors declare that the research was conducted in the absence of any commercial or financial relationships that could be construed as a potential conflict of interest.

## Publisher's Note

All claims expressed in this article are solely those of the authors and do not necessarily represent those of their affiliated organizations, or those of the publisher, the editors and the reviewers. Any product that may be evaluated in this article, or claim that may be made by its manufacturer, is not guaranteed or endorsed by the publisher.
